# Ipsilateral Patellar Tendon Reconstruction Using Quadriceps Tendon-Patella Bone Autograft for Simultaneous Rupture of the Patellar Tendon, Anterior Cruciate Ligament, and Medial Collateral Ligament: A Case Report

**DOI:** 10.7759/cureus.86465

**Published:** 2025-06-20

**Authors:** Yuki Yamanashi, Takuya Okamoto, Yusuke Morishita, Nobunori Takahashi, Masataka Deie

**Affiliations:** 1 Department of Orthopedic Surgery, Aichi Medical University Hospital, Nagakute, JPN; 2 Department of Rehabilitation Medicine, Aichi Medical University Hospital, Nagakute, JPN; 3 Department of Orthopedic Surgery, Hiroshima City Hiroshima Citizens Hospital, Hiroshima, JPN

**Keywords:** acl tear, ligament reconstruction, mcl, patellar tendon rupture, quadriceps tendons

## Abstract

Simultaneous rupture of the patellar tendon (PT), anterior cruciate ligament (ACL), and medial collateral ligament is a relatively rare type of trauma. We present a two-stage treatment for this type of trauma.

A 47-year-old male who fell from a height of 2 m. The ruptured part of the PT was in poor condition and required strong fixation; therefore, we reconstructed the tendon using quadriceps tendon-patellar bone (QTB) autograft as the first stage of the procedure. After nine months, ACL reconstruction using the semitendinosus tendon (ST) was performed as the second stage. Eighteen months after the first operation, the range of motion (ROM) was 0° of knee extension without any extension lag and 135° of flexion. Radiographs revealed that bone graft incorporation was achieved with an Insall-Salvati ratio of 1.11. The Lachman test and pivot-shift test were negative. There were no postoperative complications, including retear, loss of ROM, or infection.

The use of QTB autografts for the rupture of the PT has several advantages. First, the enthesis can be reconstructed with healthy tissue, and the PT length can be maintained. Second, autografts can be expected to be incorporated. Third, the ST can be preserved for ACL reconstruction without invading the contralateral leg.

## Introduction

Patellar tendon (PT) rupture is an uncommon injury, with an incidence of 0.68 per 100,000 person-years in the general population [1]. While isolated ACL and MCL injuries are more common, the simultaneous rupture of the PT, anterior cruciate ligament (ACL), and medial collateral ligament (MCL) is extremely rare. Although recent systematic reviews have addressed this rare type of injury, the number of reported cases remains limited, and no consensus has yet been reached regarding a definitive treatment algorithm [2,3]. Specifically, it remains unclear whether such injuries should be managed in a single-stage or staged approach, and no established guidelines exist for the optimal choice of grafts for repair or reconstruction. Notably, while previous reports have described repair techniques for PT rupture, none have documented the use of the quadriceps tendon-patellar bone (QTB) as a graft source.

We present a case of concomitant PT, ACL, and MCL rupture that was successfully treated with a two-stage surgical treatment using only an ipsilateral graft. To minimize the risk of complications and ensure a reliable recovery, a staged approach was selected. The purpose of this report is to present and share this rare case and our reconstruction technique.

This study was previously presented in poster format at the 17th Association France Japon d’Orthopédie (AFJO), held on June 13-15, 2024.

## Case presentation

History of present trauma

**Figure 1:**
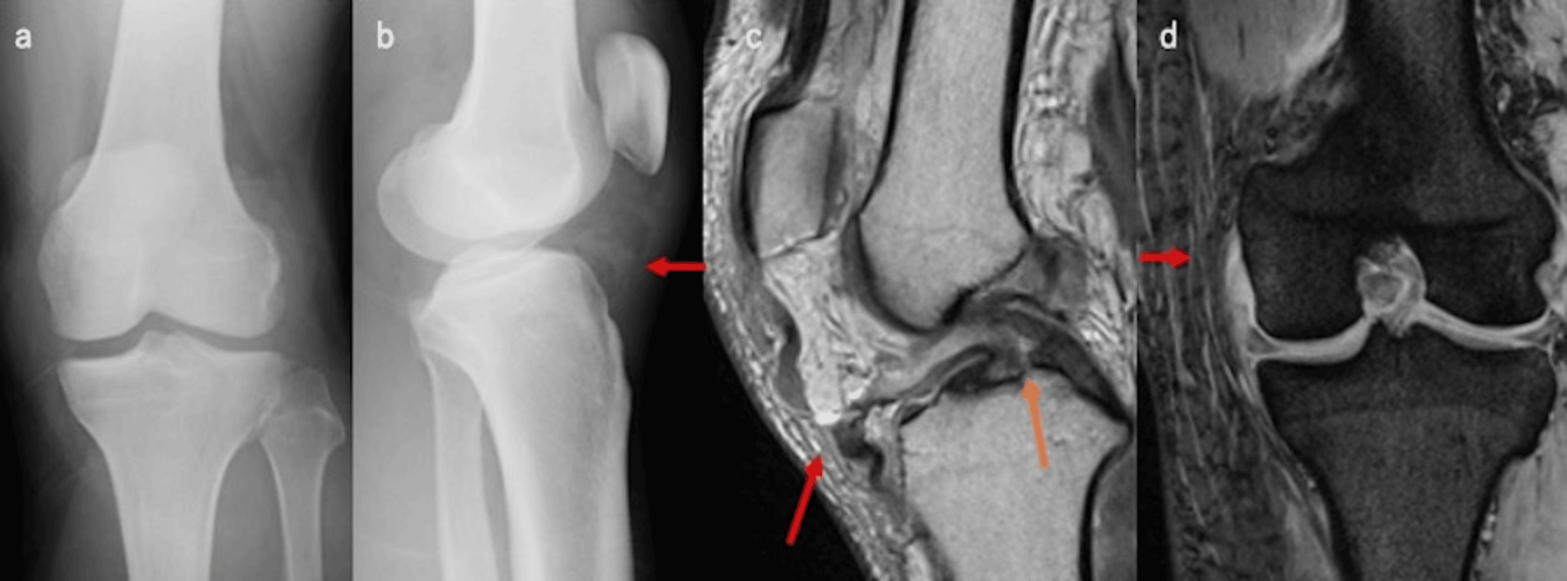
Preoperative radiograph and MRI findings (a, b) The Insall–Salvati ratio was 1.2. (c) MRI revealed a PT rupture at the mid-substance level (indicated by the red arrow) and an ACL rupture at the mid-portion (indicated by the orange arrow). (d) MCL rupture was observed at the femoral attachment site. MRI: magnetic resonance imaging, ACL: anterior cruciate ligament, MCL: medial collateral ligament, PT: patellar tendon

A 47-year-old male with no relevant medical history fell from a height of 2 m and sprained his left knee. On physical examination, the knee had severe joint effusion. The patient was unable to walk due to pain, and the range of motion (ROM) was limited to 0° of extension and 100° of flexion. The Active extension was not possible. The Lachman test and the valgus stress test performed in full extension were positive. Radiography revealed that the patellar position was slightly high and that the Insall-Salvati ratio was 1.2. MRI showed that the PT at the middle level, the mid-portion of the ACL, and the MCL were torn (Figure 1a-1d).

Moreover, the lateral meniscus (LM) was dislocated. We diagnosed the patient with a simultaneous rupture of the PT, ACL, and MCL in addition to an LM tear. Therefore, we performed the first stage of the operation eight days after the injury.

First stage of the operation

**Figure 2:**
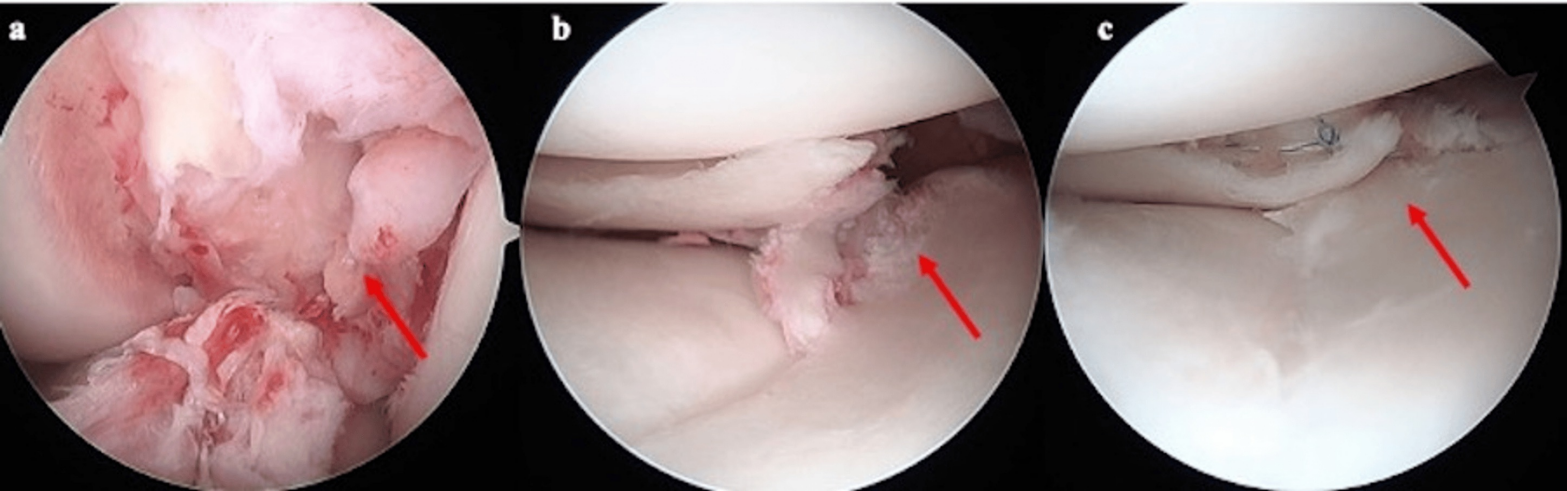
Arthroscopic examination (a) ACL tear. (b) LM radial tear. (c) LM was treated with inside-out and all-inside suture techniques. ACL: anterior cruciate ligament, LM: lateral meniscus

After arthroscopic examination, the LM was treated with inside-out and all-inside suture techniques for radial tears using arthroscopy (Figure 2a-2c).

**Figure 3:**
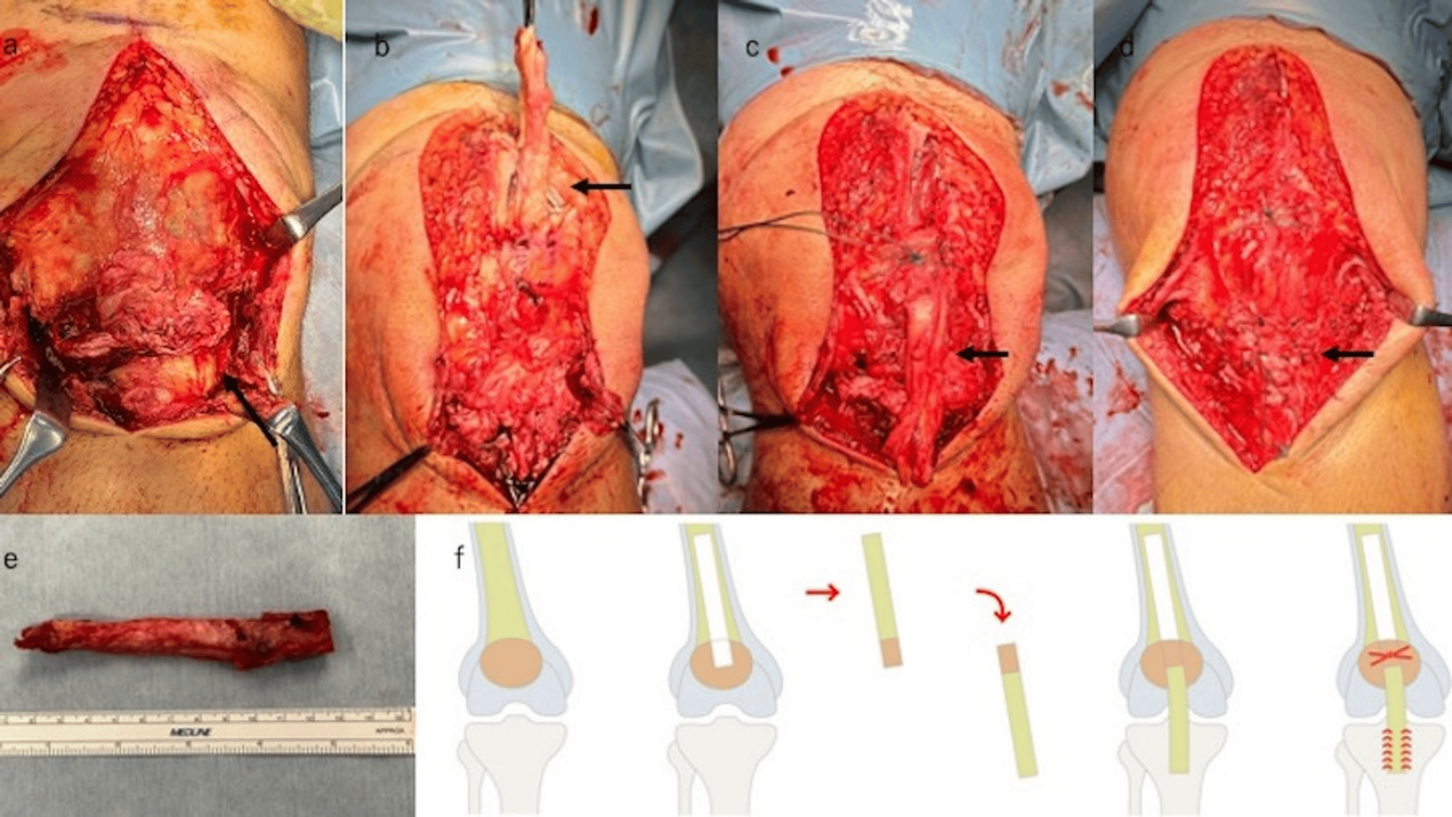
Intraoperative findings at the first stage (a) PT rupture at the middle level. The ruptured part was in poor condition and irreparable. (b, e) An 8.0 × 1.5 cm quadriceps tendon with a 2.0 × 1.5 cm patellar bone was harvested. (c, d) The tendon side was sutured with a Krackow suture by #2 Ethibond, and the end of the suture was passed through the tibial bone tunnel. The tendon side and distal injury site were sutured together using #0 Surgilon. (f) Schema of surgical treatment. Image created by Yuki Yamanashi. PT: patellar tendon

The ruptured part of the PT was in poor condition and required strong fixation for return to heavy labor; therefore, we chose to perform reconstruction using QTB autograft. In this technique, an 8.0 × 1.5 cm quadriceps tendon with a 2.0 × 1.5 cm patellar bone was harvested first. After holes were made in the patellar and tibial tuberosity for fixation, the graft was flipped upside down and fixed at the harvested location. The bone site was fixed with #5 Ethibond (Ethicon, Raritan, NJ) sutures. The tendon side was sutured with a Krackow suture with #2 Ethibond, and the end of the suture was passed through the tibial bone tunnel. A transverse bone hole was created at the tibial tuberosity, and one end of the suture was passed through it and tied manually. Fixation was performed at 45° of knee flexion, with appropriate tension to match the patellar height of the contralateral side and to avoid overtightening at the fixation site. Additionally, the tendon side and distal injury site were sutured together using #0 Surgilon (Covidien, Japan) (Figure 3a-3f).

Finally, the MCL was repaired using a JuggerKnot (Zimmer-Biomet, Warsaw, IN). Postoperative rehabilitation was performed as described below. Partial weight-bearing and ROM exercises were started two weeks after surgery. Flexion of up to 90° was allowed for the first four weeks postoperatively. Full weight-bearing walking and flexion over 120° were allowed with a hard brace six weeks after surgery.

Second stage of the operation

**Figure 4:**
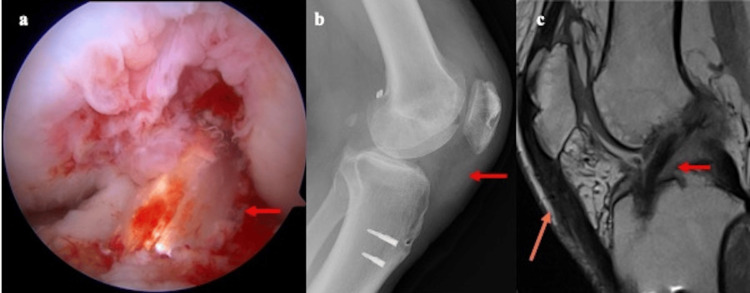
Intraoperative finding and postoperative images (a) Single bundle ACL reconstruction using the ST. (b) Postoperative radiograph. Insall-Salvatti ratio: 1.1. (c) Postoperative MRI. Reconstructed PT (indicated by the red arrow) and ACL (indicated by the orange arrow) had good continuity and density. ACL: anterior cruciate ligament, MRI: magnetic resonance imaging, PT: patellar tendon, ST: semitendinosus tendon

After full ROM was recovered, the patient was able to walk without a cane, and bone graft incorporation was achieved at nine months after the first operation. The second stage of the surgery, a single-bundle ACL reconstruction, was subsequently performed. At this time, the healing of LM was confirmed. During this operation, the ipsilateral semitendinosus tendon (ST) was harvested for grafting. The femoral side of the bone tunnel was made using the far medial technique. A drill guide was placed on the tibial side of the bone tunnel via anteromedial portals at the center of the footprint. Finally, the graft was passed through both tunnels and fixed at a 30° knee flexion position, and 30 N was applied using an Endobutton CL Ultra fixation button (Smith and Nephew, Andover, MA) on the femoral side and LP staples (Meira, Nagoya, Japan) on the tibial side (Figure 4a-4c).

Partial weight-bearing and ROM exercises were initiated one week after surgery for postoperative rehabilitation. Weight-bearing ability and ROM gradually increased every week.

Postoperative outcomes and follow-up

At 18 months after the initial operation, the patient was able to walk and run without the aid of a cane or brace. The ROM of knee extension was 0° without any extension lag, and flexion was 135°. The ROM of the contralateral side was 0° in extension and 140° in flexion. Postoperative quadriceps strength, as assessed by manual muscle testing, was graded as 5-. The results of the Lachman test, pivot-shift test, and valgus stress test were negative. Radiographs showed that the Insall-Salvati ratio was 1.1 (normal range: 0.8-1.2). The Lysholm score improved from 12 points preoperatively to 80 points postoperatively. The Tegner activity score increased from 3 preoperatively to 4 postoperatively. There were no postoperative complications, including retearing, loss of ROM, or infection.

## Discussion

In this report, we presented a relatively rare trauma case of simultaneous PT, ACL, and MCL rupture. We performed a two-stage operation. Using the ipsilateral QTB autograft for PT reconstruction and MCL repair as the first-stage procedure and the ST for ACL reconstruction as the second-stage procedure produced good clinical results with no complications.

In general, primary repair via the transosseous or anchor technique is recommended for acute PT tear [4,5]. To the best of our knowledge, most previous reports on the treatment of PT tears with concomitant ACL tears have recommended primary repair [2]. However, the ruptured part of the PT was in poor condition and was irreparable in this case. Therefore, we opted to perform PT reconstruction as the primary treatment.

Many surgical techniques for PT reconstruction have been previously reported, including one- or two-stage reconstructions, contralateral bone-tendon-bone grafts, ipsilateral ST grafts, and artificial ligament grafts [5-7]. In this case, considering the possibility of stage 2 ACL reconstruction and limited graft selection, we performed PT reconstruction using the ipsilateral QTB, which was flipped upside down and fixed in the harvested location. We believe that the use of the QTB for PT reconstruction has several advantages. First, the enthesis can be reconstructed with healthy tissue, and the PT length can be maintained. The QTB allows for graft harvesting of the necessary length without affecting the patellar height. Moreover, reliable and strong fusion of the native tissue with a reduced risk of patellar fracture can be expected. Second, autografts can be expected to be incorporated. Although artificial ligaments carry risks such as infection, skin necrosis, and material breakage, this surgical technique enables these risks to be minimized [5,8]. Third, the ST can be preserved for ACL reconstruction without invading the contralateral leg. The lack of invasion to the contralateral side is believed to have contributed to favorable functional recovery. Fourth, the use of allografts is challenging due to the limited number of donors, making them difficult to obtain in our country. Although the QTB graft is harvested from the extensor mechanism, which may potentially decrease knee extensor strength in the setting of a concomitant PT injury, the quadriceps manual muscle testing in this case improved to 5-. Furthermore, the postoperative Lysholm score substantially enhanced from 12 to 80 points. Previous reviews have reported postoperative Lysholm scores of 86-92 points following PT repair and ACL reconstruction, suggesting that the outcome achieved with our surgical technique is comparable to those reported in the literature [3].

One- or two-stage operations for concomitant PT and ACL rupture have remained a topic of controversy. Scrivano et al. reviewed a total of 21 studies and 32 patients with complete ACL and PT tears in the ipsilateral knee [9]. Eighteen patients (56.3%) underwent one-stage surgery, and 14 patients (43.7%) underwent two-stage surgery. The advantages and disadvantages of one-stage and two-stage surgery were discussed. One-stage surgical treatment offers the benefits of a single surgical intervention, resulting in overall shorter rehabilitation and a faster return to the pre-injury level of activity.

On the other hand, two-stage surgical treatment provides the advantages of two dedicated rehabilitation protocols. Some previous reports have shown that the one-stage group has complications such as persistent patellofemoral crepitus and arthrofibrosis [10,11]. These complications are associated with a risk of additional intervention or some dysfunction in the knee joint. Previous reviews have shown that ACL reconstruction is usually performed three to six months after PT reconstruction [9]. In our case, although there was a combination of irreparable PT and MCL injuries, the initial treatment was limited to PT reconstruction and MCL repair. Moreover, we started active rehabilitation two weeks after surgery. However, it took approximately six months for the ROM to recover, and we were able to perform the second stage of the operation eight months after the first stage. There is no clear evidence for these traumas, and further study is needed. We believe that aiming for definite improvement in the ROM through two-stage surgery is important. For concomitant PT and ACL rupture, we recommend treating the PT first. After recovery of ROM and muscle strength, we perform ACL reconstruction to prevent complications.

## Conclusions

We present a case of a concomitant PT, ACL, and MCL tear that was successfully treated with a two-stage surgical approach. Using the ipsilateral QTB for PT reconstruction and MCL repair as the first stage and the ST for ACL reconstruction as the second stage, good clinical outcomes were achieved without complications. Although no standardized treatment protocol exists for this rare combination of injuries, the use of the QTB graft provided a robust and biologically favorable reconstruction. Furthermore, performing the two-stage surgery allowed for safe management of the injuries without any postoperative complications. This technique may serve as a viable treatment option in cases where particularly strong fixation is required.

## References

[1] Chen SK, Lu CC, Chou PH, Guo LY, Wu WL (2009). Patellar tendon ruptures in weight lifters after local steroid injections. Arch Orthop Trauma Surg.

[2] O'Dowd JA, Lehoang DM, Butler RR, Dewitt DO, Mirzayan R (2020). Operative treatment of acute patellar tendon ruptures. Am J Sports Med.

[3] Ismailidis P, Neopoulos G, Egloff C (2024). Simultaneous patellar tendon and anterior cruciate ligament rupture: a systematic review, meta-analysis and algorithmic approach. Arch Orthop Trauma Surg.

[4] Tandogan RN, Terzi E, Gomez-Barrena E, Violante B, Kayaalp A (2022). Extensor mechanism ruptures. EFORT Open Rev.

[5] Matthews AH, Fraser EJ, Parkinson B (2018). Management of simultaneous patellar tendon and anterior cruciate ligament ruptures-a systematic review of available literature. J Orthop Trauma.

[6] Gilmore JH, Clayton-Smith ZJ, Aguilar M, Pneumaticos SG, Giannoudis PV (2015). Reconstruction techniques and clinical results of patellar tendon ruptures: evidence today. Knee.

[7] Saragaglia D, Pison A, Rubens-Duval B (2013). Acute and old ruptures of the extensor apparatus of the knee in adults (excluding knee replacement). Orthop Traumatol Surg Res.

[8] Wood TJ, Leighton J, Backstein DJ (2019). Synthetic graft compared with allograft reconstruction for extensor mechanism disruption in total knee arthroplasty: a multicenter cohort study. J Am Acad Orthop Surg.

[9] Scrivano M, Ticca L, Pasquale Vadala A, Fedeli G, Rossato A, Ferretti A (2022). A different unhappy triad in the knee: a case of acute simultaneous rupture of patellar tendon, anterior cruciate ligament and lateral meniscus treated in one stage and review of literature. Orthop Rev (Pavia).

[10] Futch LA, Garth WP, Folsom GJ, Ogard WK (2007). Acute rupture of the anterior cruciate ligament and patellar tendon in a collegiate athlete. Arthroscopy.

[11] Levakos Y, Sherman MF, Shelbourne KD, Trakru S, Bonamo JR (1996). Simultaneous rupture of the anterior cruciate ligament and the patellar tendon. Six case reports. Am J Sports Med.

